# Report of the King's Fund Distribution Committee

**Published:** 1914-12-26

**Authors:** 


### Report of the King's Fund Distribution Committee.

### ADOPTED AT THE MEETING OF THE GOVERNORS AND GENERAL COUNCIL.

(1) The Governors and General Council have this year fixed the sum available for distribution amongst the London hospitals at £133,500, as compared with £151,000 distributed in 1913.

(2) The Distribution Committee have, nevertheless, been able to recommend an increase in the total grants to maintenance. This course, which the committee think will prove to be the most effective way of helping the hospitals in their present circumstances, has been rendered possible by the fact that, in view of the war, the hospitals have, with the approval of the Fund, deferred all schemes of building or improvement that were not already in hand or particularly urgent; with the result that the demands on the Fund for contributions in aid of capital expenditure have been considerably reduced. The total of grants to buildings and improvements, which last year was £35,650, is this year £12,350; while the total of the grants recommended to maintenance, including grants in reduction of maintenance debts, amounts to £121,150, as against £115,350 last year.

(3) Although there has been an increase in the total amount recommended for maintenance grants, and a decrease in the total for capital grants, the individual awards vary, as compared with last year, in accordance with the particular circumstances of each separate hospital, including the financial position of each institution as disclosed by its income and expenditure account and balance-sheet for 1913, the last completed year. Moreover, in the case of all applications, whether for maintenance grants or for grants in reduction of debt or in aid of building schemes, the committee have felt obliged this year to pay special attention to the question of relative urgency.

It must not be assumed, therefore, that a reduction in the grant of any particular institution, whether for maintenance or for capital purposes, or even the absence of a grant, implies dissatisfaction.

(4) Although the Fund has applied for and obtained from the hospitals information on their financial position up to as late a date as possible for the current year before the committee considered the applications made to it, the examination of the relative needs of the various institutions has been necessarily based for the most part on the last completed and audited accounts—namely, those for the year 1913. But the effect of the first few months of the war on the statistics and finances of the hospitals, as well as the probable effect of their action in placing beds at the disposal of the naval and military authorities during the present emergency, has not been overlooked. The result of these new conditions will be reflected in the accounts for 1914, and will thus form part of the material submitted to the Fund in connection with next year's applications.

(5) The number of hospitals applying for grants is 104, as against 106 last year. This reduction is due to the successful completion of two schemes of amalgamation.

(6) The amalgamation of the Hospital for Diseases of the Throat, Golden Square, and the London Throat Hospital, Great Portland Street, was reported to the General Council at the annual meeting in April last, when a capital grant of £10,000, with a maintenance grant of £900 a year for five years, subject to the usual annual inspection, was approved. Out of the moneys set aside or promised towards a general scheme of amalgamation of the Throat, Nose, and Ear Hospitals, there still remain unallocated a capital sum of £10,500, and maintenance grants not exceeding £1,100 a year. The Distribution Committee recommend that power be given to them to draw upon the balance of the capital and maintenance grants so promised or set aside, in such proportions as they may consider appropriate, in the event of any further amalgamation scheme being brought to a satisfactory conclusion at a time when a meeting of the Governors and General Council is not pending.

(7) The committee are glad to report that the proposed amalgamation between University College Hospital and the National Dental Hospital has been brought into effect during the year. The latter hospital is now the dental department of the former. In recommending the amount of the grant to University College Hospital, the Distribution Committee have taken into account the work done both while the National Dental Hospital existed as a separate institution and since it has become a branch of University College Hospital.

(8) The only grant made for a capital purpose in 1912 which remains unapplied, and which therefore comes up for reconsideration under the two years' rule, is one of £500 to the provision of a new operating theatre at the West London Hospital. The committee are informed that the scheme to which the grant was made has been abandoned in favour of a larger scheme of improvements, which is still under consideration. They recommend, therefore, that the grant made in 1912 to the smaller scheme should lapse, and that the fresh proposals, when received in their final form, should be considered as a new scheme.

(9) In dealing with the applications of the various hospitals, the committee has, as usual, taken into consideration not only relative need, but also efficiency and economy as revealed by the evidence before them, including the visitors' reports and the statistics of work done and cost of working. Where the figures in the Fund's Statistical Report indicate an excess of cost, above the average of comparable hospitals, greater than appears to the committee to be justified by any special features in the work or circumstances of the institution, the committee recommend that the attention of the authorities of the hospital should be called to the fact. In some cases they recommend that the committee of the hospital should set on foot a systematic inquiry into the question with a view to effecting economies. The Distribution Committee recognise that at the present moment there are exceptional factors tending unavoidably to raise the cost of hospital maintenance, but for this very reason it appears to them most important that every care should be taken to strengthen the control over expenditure wherever the cost of working in normal times affords evidence that this is needed.

(10) The visitors' reports have again proved of the utmost value, and the committee are greatly indebted to the medical men and laymen who undertake this work. The annual inspection makes greater demands on the time of the visitors now than it did when the area of operations of the Fund was smaller, and when there were therefore fewer suburban hospitals on the list. The committee have continued the practice of supplementing their awards by recommending that extracts from the visitors' reports should be communicated privately to the hospitals concerned, a course which has proved very beneficial in the past.

(11) The committee welcome the publication of the new edition of the Revised Uniform System of Hospital Accounts, incorporating the decisions of the three Funds upon all the questions which have arisen out of the application of the system to the accounts of the various hospitals. The Distribution Committee much appreciate the co-operation of the Metropolitan Hospital Sunday Fund and the Hospital Saturday Fund, and of the Committee of Hospital Secretaries, in the work of revision, the result of which has been greatly to lighten during the last few years the labour of comparing the accounts and statistics of the institutions applying for grants. They are also grateful to the hospital officials and auditors for the time and trouble spent in applying the system, and for many valuable suggestions towards the completion of the work.

The details of the awards to the various hospitals are appended.

